# Association Between Blood Transfusion Volume and Adverse Clinical Outcomes in Chronic Liver Disease: A Retrospective Observational Study

**DOI:** 10.7759/cureus.115857

**Published:** 2026-09-06

**Authors:** Aditya Saha, Akshay Saha, Neelam Shah

**Affiliations:** 1 General Medicine, Kokrajhar Medical College and Hospital, Kokrajhar, IND; 2 Nephrology, Institute of Post Graduate Medical Education and Research, Kolkata, IND; 3 Ophthalmology, Employees State Insurance Post Graduate Institute of Medical Sciences and Research, Joka, IND

**Keywords:** chronic liver disease (cld), hepatic encephalopathy (he), model for end-stage liver disease (meld) score, portal hypertension, restrictive blood transfusion, variceal rebleeding

## Abstract

Background

Blood transfusion is frequently required in patients with chronic liver disease, particularly during episodes of gastrointestinal bleeding. However, excessive transfusion may increase portal venous pressure and adversely affect clinical outcomes. This study aimed to evaluate the association between transfusion volume and adverse clinical outcomes in patients with chronic liver disease.

Objective

This study aimed to assess the association between blood transfusion volume and adverse clinical outcomes, including rebleeding, hepatic encephalopathy, ascites, and prolonged hospital stay, among patients with chronic liver disease.

Methods

This retrospective observational study was conducted at Assam Medical College, Dibrugarh, Assam, India, and utilized existing patient records collected between April 1, 2025, and March 31, 2026. A total of 240 adult patients with chronic liver disease who received blood transfusion during hospitalization were included. Patients were categorized into low-transfusion (≤2 units) and high-transfusion (>2 units) groups. Outcomes assessed included rebleeding, hepatic encephalopathy, ascites, and length of hospital stay. Categorical variables were analyzed using the chi-squared test, while continuous variables were compared using Student's t-test. Multivariate logistic regression analysis was performed to identify the independent predictors of rebleeding after adjustment for clinically relevant covariates.

Results

One hundred and thirty-eight (57.5%) received ≤2 units of blood transfusion, while 102 patients (42.5%) received >2 units. Rebleeding occurred more frequently in the high-transfusion group compared with the low-transfusion group (42 (41.2%) vs. 27 (19.6%); p<0.001). Hepatic encephalopathy was also more common among patients receiving >2 units (45 (44.1%) vs. 36 (26.1%); p=0.003). The mean hospital stay was significantly longer in the high-transfusion group (8.6±2.3 days vs. 5.9±1.8 days; p<0.001). On multivariate logistic regression analysis, transfusion volume >2 units independently predicted rebleeding (OR: 2.3; 95% CI: 1.1-4.8; p=0.02).

Conclusions

Higher transfusion volume was associated with increased rates of rebleeding, hepatic encephalopathy, and prolonged hospital stay in patients with chronic liver disease. These findings support careful transfusion practices and highlight the need for further prospective studies evaluating restrictive transfusion strategies.

## Introduction

Chronic liver disease is frequently associated with anemia and gastrointestinal bleeding, often necessitating blood transfusion during hospitalization. Portal hypertension, coagulopathy, hypersplenism, nutritional deficiencies, and recurrent variceal hemorrhage contribute significantly to anemia in patients with cirrhosis and chronic hepatic dysfunction [1]. Although blood transfusion remains an essential life-saving intervention, excessive transfusion may increase portal venous pressure and potentially precipitate recurrent gastrointestinal bleeding [2].

Restrictive transfusion strategies have gained increasing attention in the management of cirrhotic patients with acute gastrointestinal hemorrhage. Previous studies have demonstrated improved clinical outcomes with conservative transfusion thresholds compared with liberal transfusion practices in upper gastrointestinal bleeding [3]. Increased intravascular volume following larger transfusion volumes may influence portal pressure and has been proposed as a potential mechanism underlying associations with variceal rebleeding and hepatic decompensation; however, the present observational study cannot establish such a mechanism.

Despite growing evidence supporting restrictive transfusion practices, limited real-world observational data are available regarding the relationship between transfusion volume and clinical outcomes among patients with chronic liver disease in routine clinical settings. Therefore, the present study aimed to evaluate the association between blood transfusion volume and adverse clinical outcomes, including rebleeding, hepatic encephalopathy, ascites, and duration of hospital stay, among patients with chronic liver disease admitted to a tertiary care center.

## Materials and methods

Study design and setting

This retrospective observational study was conducted at Assam Medical College, Dibrugarh, a leading tertiary care center in Assam, India, using existing patient records from eligible patients admitted to the Department of General Medicine in the time frame from April 1, 2025, to March 31, 2026.

Ethical approval

The study was approved by the Institutional Ethics Committee (H) of Assam Medical College, Dibrugarh (approval number: AMC/EC/2025/1799) and was conducted in accordance with the Declaration of Helsinki. This study was reported in accordance with the Strengthening the Reporting of Observational Studies in Epidemiology (STROBE) reporting guideline [4].

Study population

A total of 240 consecutive adult patients with chronic liver disease who were admitted during the study period and received blood transfusion during hospitalization were included in the study. The diagnosis of chronic liver disease was established from documented medical records, based on a compatible clinical history together with relevant laboratory, imaging, and/or endoscopic findings indicating chronic hepatic dysfunction or cirrhosis. Patients were categorized into a lower-transfusion group (≤2 units) and a higher-transfusion-burden group (>2 units). This pre-specified cut-off was used as a pragmatic analytical categorization to distinguish patients receiving limited transfusion support from those with greater transfusion exposure. The cut-off was not intended to represent a validated clinical or biological threshold, and the >2-unit group was not considered to represent a homogeneous level of transfusion exposure or a specific dose-response category. The selection of eligible subjects is shown in Figure 1.

**Figure 1:**
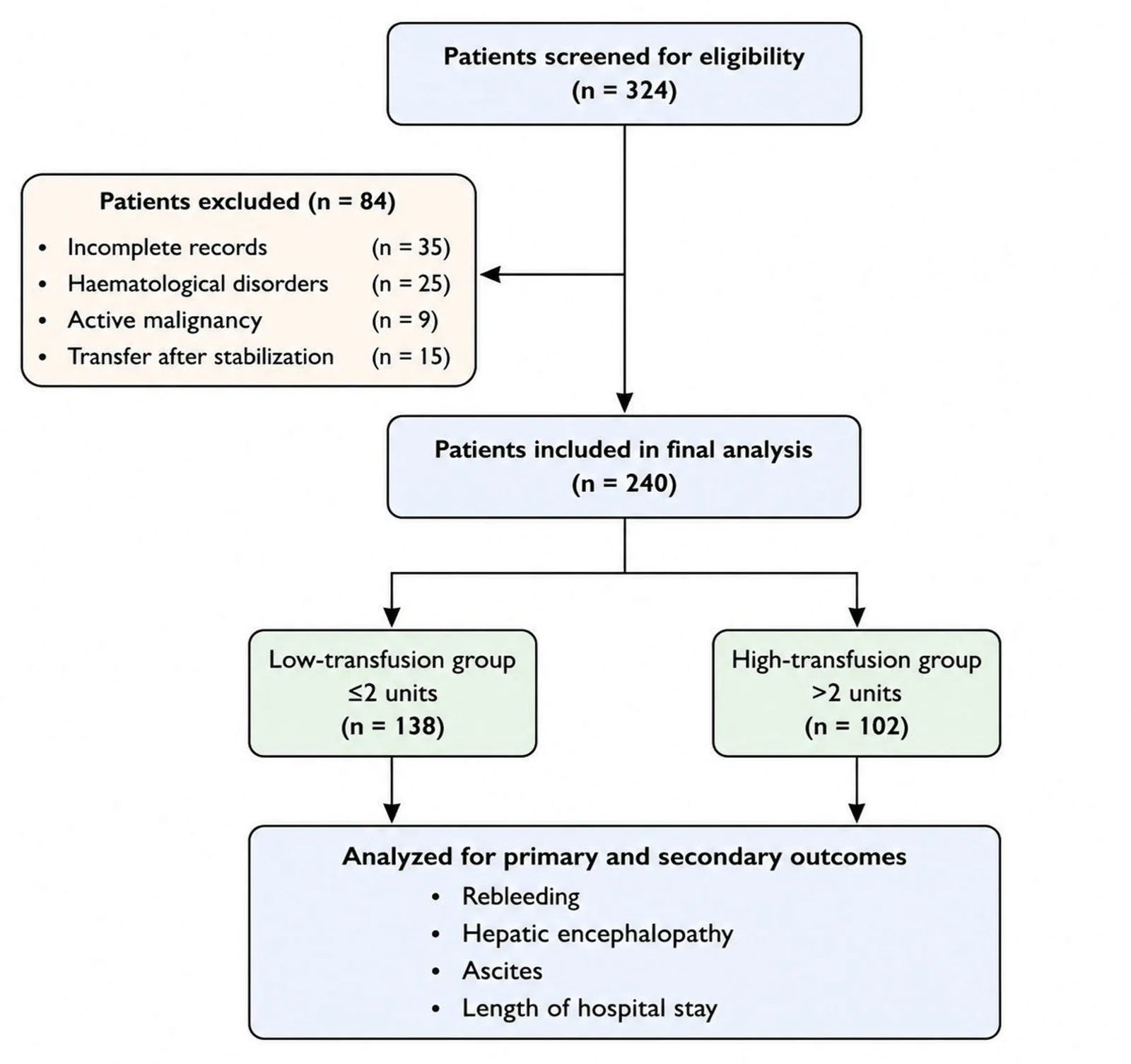
Flowchart depicting patient selection and study enrollment

Inclusion criteria

Adult patients (≥18 years) with documented chronic liver disease, established from a compatible clinical history together with relevant laboratory, imaging, and/or endoscopic findings indicating chronic hepatic dysfunction or cirrhosis, who received blood transfusion during hospitalization were eligible for inclusion.

Exclusion criteria

Patients with incomplete medical records, active malignancy, or hematological disorders unrelated to chronic liver disease or those transferred from other institutions after initial stabilization were excluded from the study.

Transfusion exposure

The transfusion exposure analyzed in this study consisted of packed red blood cells. The number of transfused units was obtained from the documented hospital transfusion records. Transfusion volume was defined as the total number of units administered during the hospitalization, including units administered after a documented episode of rebleeding. Patients were categorized into a lower-transfusion group (≤2 units) and a higher-transfusion-burden group (>2 units). This pre-specified categorization was used for pragmatic analytical comparison and was not intended to represent a validated clinical transfusion threshold or a dose-response relationship. Transfusion decisions were made by the treating clinical team according to the patient's clinical condition, severity of bleeding, hemodynamic status, and hemoglobin level. No study-specific transfusion threshold was imposed because of the retrospective observational design.

Outcome measures

Primary Outcome

Rebleeding was defined as recurrent hematemesis, melena, or a decrease in hemoglobin level of ≥2 g/dL after initial stabilization requiring repeat resuscitation, blood transfusion, endoscopic intervention, or hospital readmission during the same hospitalization period [5].

Secondary Outcomes

Secondary outcome measures included hepatic encephalopathy, ascites, and length of hospital stay. Hepatic encephalopathy was diagnosed clinically according to the West Haven criteria [6]. Ascites was diagnosed based on clinical examination and/or radiological evidence. Length of hospital stay was defined as the total duration from hospital admission to discharge and was recorded in days. These outcomes were selected because they represent important indicators of hepatic decompensation and healthcare utilization in patients with chronic liver disease.

Statistical analysis

Statistical analysis was performed using IBM SPSS Statistics for Windows, Version 26.0 (IBM Corp., Armonk, New York, United States). Continuous variables were assessed for normality before analysis. Categorical variables were analyzed using the chi-squared test, while continuous variables were compared using Student's t-test.

Multivariable logistic regression analysis was performed to assess factors independently associated with rebleeding. The Model for End-Stage Liver Disease (MELD) score, etiology of chronic liver disease, and baseline hemoglobin level were included as clinically relevant covariates because they may reflect underlying liver disease severity and the clinical circumstances associated with transfusion requirement and rebleeding.

Patients with missing key exposure, clinical, or outcome variables were excluded from the corresponding analysis. No imputation of missing values was performed. A two-tailed p-value of <0.05 was considered statistically significant.

## Results

Baseline characteristics

A total of 240 patients were included in the study. Of these, 138 patients (57.5%) received ≤2 units of transfusion, while 102 patients (42.5%) received >2 units. Patients in the higher-transfusion-burden group had significantly higher MELD scores and lower baseline hemoglobin levels than those in the lower-transfusion group. The >2-unit category represented a range of transfusion exposure and was used for analytical comparison rather than to imply equivalence among individual transfusion volumes. Baseline characteristics of the study population are summarized in Table 1.

**Table 1:** Baseline characteristics

Variable	≤2 units (n=138)	>2 units (n=102)	Test	Statistic	P-value
Age (years)	53.6±11.4	56.8±10.2	Student's t-test	t=1.3	0.194
Male sex	87 (63%)	72 (70.6%)	Chi-squared test	χ²=1.49	0.22
Model for End-stage Liver Disease score	14.2±4.6	21.8±5.9	Student's t-test	t=-10.81	<0.001
Hemoglobin (g/dL)	10.4±1.6	8.1±1.3	Student's t-test	t=12.27	<0.001
Alcohol-related chronic liver disease	54 (39.1%)	48 (47.1%)	Chi-squared test	χ²=1.51	0.22

Clinical outcomes

Rebleeding occurred significantly more frequently in the higher-transfusion group compared with the lower-transfusion group (42 (41.2%) vs. 27 (19.6%); p<0.001). Hepatic encephalopathy was also significantly more common among patients receiving >2 units of blood transfusion (45 (44.1%) vs. 36 (26.1%); p=0.003). A statistically significant difference was observed in the prevalence of ascites between the groups (69 (67.6%) vs. 75 (54.3%); p=0.038). Clinical outcomes according to transfusion volume are summarized in Table 2.

**Table 2:** Clinical outcomes according to transfusion volume

Outcome	≤2 units (n=138)	>2 units (n=102)	Test	Statistic	P-value
Rebleeding	27 (19.6%)	42 (41.2%)	Chi-squared test	χ²=13.37	<0.001
Hepatic encephalopathy	36 (26.1%)	45 (44.1%)	Chi-squared test	χ²=8.53	0.003
Ascites	75 (54.3%)	69 (67.6%)	Chi-squared test	χ²=4.3	0.038

Length of hospital stay

The mean duration of hospital stay was significantly longer in patients receiving >2 units of blood transfusion (8.6±2.3 days) compared with those receiving ≤2 units (5.9±1.8 days), with p<0.001 (Table 3).

**Table 3:** Length of hospital stay according to transfusion volume

Variable	≤2 units (n=138)	>2 units (n=102)	Statistic	P-value
Length of hospital stay (mean±SD, days)	5.9±1.8	8.6±2.3	t=9.84	<0.001

Multivariate logistic regression analysis

Multivariable logistic regression analysis demonstrated that transfusion volume >2 units was independently associated with rebleeding (OR: 2.3; 95% CI: 1.1-4.8; Wald χ²=4.91; p=0.02). MELD score was also independently associated with rebleeding (OR: 1.09; 95% CI: 1.02-1.17; Wald χ²=6.06; p=0.01). Baseline hemoglobin <7 g/dL was not significantly associated with rebleeding (Table 4).

**Table 4:** Multivariate logistic regression analysis for rebleeding

Variable	OR	95% CI	Wald χ²	P-value
>2-unit transfusion	2.3	1.1-4.8	4.91	0.02
Model for End-stage Liver Disease score	1.09	1.02-1.17	6.06	0.01
Hemoglobin <7 g/dL	1.6	0.8-3.2	1.77	0.14

## Discussion

The present retrospective observational study found that patients with chronic liver disease receiving more than two units of packed red blood cells had significantly higher rates of rebleeding, hepatic encephalopathy, ascites, and prolonged hospitalization. Moreover, transfusion volume >2 units remained independently associated with rebleeding after adjustment for MELD score, etiology of chronic liver disease, and baseline hemoglobin level. These findings suggest that higher transfusion burden is associated with adverse clinical outcomes in patients with chronic liver disease, although the observed associations may also reflect differences in underlying disease severity and bleeding severity [3,7-9].

Although blood transfusion is essential in the management of acute gastrointestinal bleeding, liberal transfusion may increase portal venous pressure, impair clot stability, and predispose patients to recurrent hemorrhage [8-11]. Accordingly, restrictive transfusion strategies have become a cornerstone of acute variceal bleeding management and are recommended by major international guidelines [9,12-14].

The association between higher transfusion volume and increased rebleeding in the present study is consistent with previous evidence supporting restrictive transfusion strategies in acute upper gastrointestinal bleeding. In the landmark randomized trial by Villanueva et al., a restrictive transfusion strategy (hemoglobin threshold of 7 g/dL) reduced further bleeding and improved survival, particularly in patients with cirrhosis and portal hypertension [3]. These findings have been supported by subsequent studies and systematic reviews demonstrating that restrictive transfusion reduces recurrent bleeding without increasing mortality or other major adverse events [15-18]. Consistent with these reports, our study demonstrated that transfusion of more than two units remained independently associated with rebleeding after adjustment for disease severity and baseline hemoglobin.

Similarly, observational studies have reported that higher transfusion requirements are associated with poorer clinical outcomes in cirrhosis and acute gastrointestinal bleeding [16,17,19]. Although greater transfusion volume may reflect more severe bleeding, excessive transfusion can further increase portal pressure and predispose to recurrent hemorrhage [8,10]. While our findings demonstrate an independent association between higher transfusion burden and rebleeding in the multivariable model, causality cannot be established because of the retrospective design and the possibility of residual confounding.

In the present study, patients receiving more than two units of blood transfusion had significantly higher rates of hepatic encephalopathy and ascites. These findings may reflect more advanced liver disease and greater severity of acute gastrointestinal bleeding among patients requiring larger transfusion volumes. Physiological effects associated with larger transfusion volumes may also be relevant; however, their independent contribution cannot be determined from the present retrospective analysis. Previous studies have shown that patients with decompensated cirrhosis and acute variceal bleeding are at increased risk of hepatic encephalopathy because of impaired hepatic clearance, systemic inflammation, circulatory dysfunction, and increased nitrogen load from transfused blood products [8-10,12,20,21].

Similarly, the higher prevalence of ascites in the higher-transfusion-burden group may reflect more severe portal hypertension rather than a direct effect of transfusion alone. As portal hypertension drives both ascites formation and variceal hemorrhage, patients with more advanced disease are more likely to require larger transfusion volumes [8-10]. Therefore, while higher transfusion volume was associated with hepatic decompensation-related outcomes in our study, this relationship should be interpreted cautiously, as transfusion burden may represent a marker of advanced liver disease and greater clinical severity rather than an independent cause of these outcomes. Prospective studies incorporating hepatic venous pressure gradient measurements and standardized transfusion protocols are needed to clarify these associations [9,10,12].

Patients receiving more than two units of blood transfusion had significantly longer hospital stays, consistent with previous studies linking higher transfusion requirements in cirrhosis to greater disease severity, increased complications, and prolonged hospitalization [3,13,17,19]. Recurrent bleeding, hepatic encephalopathy, ascites, and the need for repeated interventions may have contributed to delayed recovery and may partly explain the longer hospital stay observed among patients receiving higher transfusion volumes. However, the retrospective design does not allow the determination of whether transfusion burden itself contributed to prolonged hospitalization [16,17].

Notably, transfusion volume >2 units remained independently associated with rebleeding after adjustment for MELD score, etiology of chronic liver disease, and baseline hemoglobin. As the MELD score is a well-established predictor of disease severity and mortality [7,8], this finding suggests that transfusion burden may be associated with rebleeding beyond the effects captured by the selected covariates. However, residual confounding by indication cannot be excluded despite multivariable adjustment. Accordingly, this association should be considered hypothesis-generating rather than evidence of a causal relationship. Patients requiring greater transfusion support may have had more severe bleeding or greater hemodynamic instability at baseline, and these factors may themselves increase the risk of rebleeding. Therefore, the observed association should be considered hypothesis-generating rather than evidence of a causal effect of transfusion volume on rebleeding [15,17,18].

The findings of the present study have important clinical implications. Although blood transfusion remains a life-saving intervention in acute gastrointestinal bleeding, the observed association between higher transfusion burden and adverse clinical outcomes underscores the importance of careful and individualized transfusion practices in patients with chronic liver disease. Decisions regarding transfusion should continue to consider bleeding severity, hemodynamic status, hemoglobin level, and the overall clinical context. Our findings should not be interpreted as establishing the superiority of a particular restrictive transfusion protocol, but rather as supporting the need for further prospective evaluation of transfusion strategies in this population [3,9,12-15]. Careful assessment of bleeding severity, hemodynamic status, and hemoglobin level is essential to optimize transfusion practices and minimize the risk of adverse outcomes [9,12-14].

Strengths

The present study has several strengths. It evaluated real-world transfusion practices in hospitalized patients with chronic liver disease using consecutive patient data and assessed clinically relevant outcomes including rebleeding, hepatic encephalopathy, ascites, and length of hospital stay. The comparison of predefined transfusion-burden groups and adjustment for clinically relevant factors, including MELD score and baseline hemoglobin, strengthen the analysis. In addition, the study provides real-world observational data regarding the relationship between transfusion burden and adverse clinical outcomes in patients with chronic liver disease.

Limitations

However, several limitations should be acknowledged. First, the retrospective single-center design and relatively modest sample size limit the generalizability of the findings and preclude definitive causal inference. Second, confounding by indication is an important limitation. Patients receiving >2 units had substantially higher MELD scores and lower baseline hemoglobin levels than those receiving ≤2 units, suggesting that the higher-transfusion group was clinically more severely ill at baseline. Although multivariable adjustment was performed for clinically relevant factors, residual confounding related to the severity of gastrointestinal bleeding, hemodynamic instability, variceal characteristics, endoscopic findings and interventions, and other unmeasured clinical factors cannot be excluded. Third, the retrospective nature of the study limits the ability to establish a clear temporal relationship between transfusion exposure and subsequent rebleeding. In particular, transfusions administered in response to an episode of rebleeding could contribute to the observed association and introduce the possibility of reverse causation, depending on the timing of exposure relative to the outcome. Fourth, detailed information regarding the clinical indications and transfusion thresholds, as well as comprehensive endoscopic characteristics and long-term follow-up, was not consistently available in the retrospective records. Mortality outcomes were also not available for analysis.

Therefore, the observed associations should be interpreted as hypothesis-generating and should not be considered evidence of a causal effect of higher transfusion volume on adverse clinical outcomes. Prospective multicenter studies with standardized transfusion protocols, clearly defined exposure windows, detailed bleeding and endoscopic characteristics, and longitudinal follow-up are needed to clarify the relationship between transfusion burden and outcomes in patients with chronic liver disease [5,17].

## Conclusions

Higher transfusion volume was associated with increased rates of rebleeding, hepatic encephalopathy, ascites, and prolonged hospitalization among patients with chronic liver disease. Although limited by its retrospective single-center design, this study supports an association between higher transfusion burden and adverse clinical outcomes in chronic liver disease. These findings reinforce the importance of careful transfusion practices but do not establish a causal relationship between transfusion volume and adverse outcomes. Larger prospective multicenter studies are needed to validate these associations and evaluate standardized transfusion strategies in this high-risk population.
